# Visual question answering model for fruit tree disease decision-making based on multimodal deep learning

**DOI:** 10.3389/fpls.2022.1064399

**Published:** 2023-01-05

**Authors:** Yubin Lan, Yaqi Guo, Qizhen Chen, Shaoming Lin, Yuntong Chen, Xiaoling Deng

**Affiliations:** ^1^ College of Electronic Engineering, College of Artificial Intelligence, South China Agricultural University, Guangzhou, China; ^2^ Guangdong Laboratory for Lingnan Modern Agriculture, Guangzhou, China; ^3^ National Center for International Collaboration Research on Precision Agricultural Aviation Pesticide Spraying Technology, Guangzhou, China; ^4^ Guangdong Engineering Technology Research Center of Smart Agriculture, Guangzhou, China

**Keywords:** disease decision-making, deep learning, multimodal fusion, visual question answer, bilinear model, co-attention mechanism

## Abstract

Visual Question Answering (VQA) about diseases is an essential feature of intelligent management in smart agriculture. Currently, research on fruit tree diseases using deep learning mainly uses single-source data information, such as visible images or spectral data, yielding classification and identification results that cannot be directly used in practical agricultural decision-making. In this study, a VQA model for fruit tree diseases based on multimodal feature fusion was designed. Fusing images and Q&A knowledge of disease management, the model obtains the decision-making answer by querying questions about fruit tree disease images to find relevant disease image regions. The main contributions of this study were as follows: (1) a multimodal bilinear factorized pooling model using Tucker decomposition was proposed to fuse the image features with question features: (2) a deep modular co-attention architecture was explored to simultaneously learn the image and question attention to obtain richer graphical features and interactivity. The experiments showed that the proposed unified model combining the bilinear model and co-attentive learning in a new network architecture obtained 86.36% accuracy in decision-making under the condition of limited data (8,450 images and 4,560k Q&A pairs of data), outperforming existing multimodal methods. The data augmentation is adopted on the training set to avoid overfitting. Ten runs of 10-fold cross-validation are used to report the unbiased performance. The proposed multimodal fusion model achieved friendly interaction and fine-grained identification and decision-making performance. Thus, the model can be widely deployed in intelligent agriculture.

## 1 Introduction

In recent years, deep learning (DL) has reached an advanced stage in computer vision and natural language processing, and multimodal learning has become a popular research topic in deep learning research. In the field of fruit tree disease research, the diagnosis and decision-making of fruit tree diseases traditionally rely on the observations of experts or experienced farmers to remove diseased plants as early as possible. Most applications of deep learning for fruit tree diseases use only single-source data, including images, spectra, and meteorological data (Wang et al., 2019; Zhan et al., 2022), mainly based on visual images for disease recognition and classification (Deng et al., 2019; Lan et al., 2020) or text-based intelligent Q&A for diseases. However, if a machine is trained to become an image analysis of diseased plants, obtaining the corresponding disease diagnosis and decision based on human questions is critical for smart agriculture. Yang et al. (2021) proposed the multimodal bilinear fusion of the Citrus Huanglongbing (HLB) detection network by fusing RGB images and hyperspectral information and achieved good results, which indicates that fusion of multi-source data information can make disease identification more accurate. Multimodal information fusion technology in smart orchards has become a current research hot spot that can solve the problem that single-source data cannot extract more fine-grained fruit tree disease information.

In traditional agriculture, the diagnosis and decision-making of fruit tree diseases depend on the observation of experts or experienced farmers to remove diseased plants as early as possible. However, in practice, the diagnosis of diseased plants first relies on experts to identify and then consult agricultural knowledge before obtaining a treatment method. Training a machine to become an image analyzer of diseased plants and obtain the corresponding disease diagnosis and decision based on human questions aligns well with smart agriculture. To further improve the performance of disease decision issues, we must combine image and text into multimodality for the Visual Question Answering (VQA) task to implement decision-making on fruit tree diseases.

**Figure 1:**
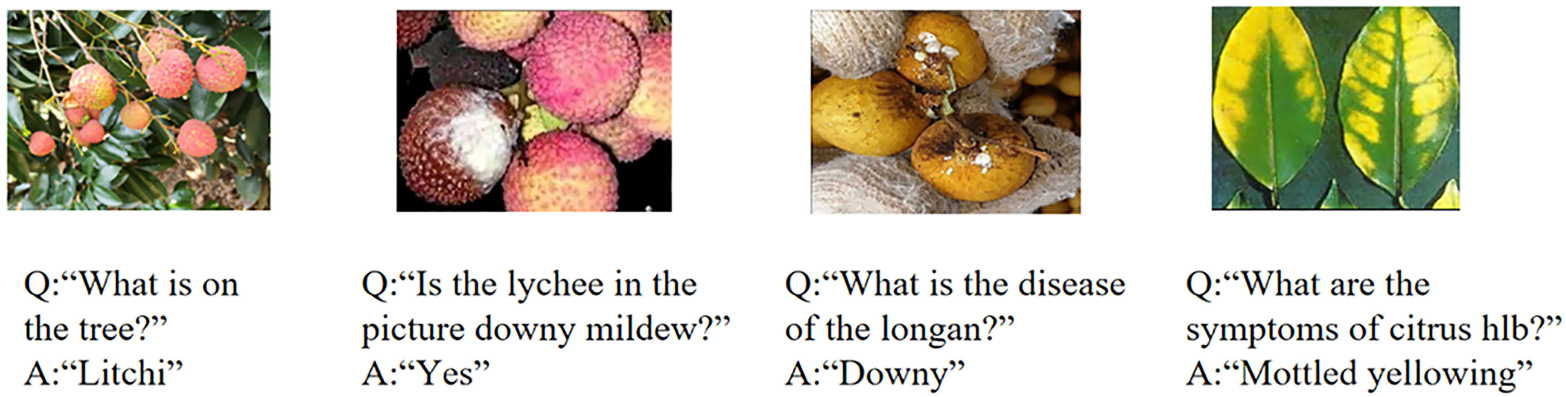
Examples of questions and images and their corresponding answers in fruit tree diseases based on VQA.

Fruit tree disease decision-making research is based on VQA in a multimodal learning task. **Figure 1** shows four examples of VQA for fruit tree diseases, which illustrates that VQA provides accurate decision-making in orchards by detecting diseases and querying the answers to questions arising from the actual environment, which is important to guide farmers to obtain timely feedback and make decisions on diseases in orchards. VQA aims to answer relevant questions based on images (Malinowski and Fritz, 2014; Antol et al., 2015). It requires a fine-grained semantic understanding of images and questions and also guides visual reasoning to predict precise answers based on the questions. Representation learning of VQA can be divided into extracting images and question features. The image feature extraction models mainly include Convolutional Neural Network (CNN), VGGNet (Simonyan and Zisserman, 2014), GoogLeNet (Szegedy et al., 2015), and ResNet (Fukui et al., 2016; Kim et al., 2016; Ben-Younes et al., 2017) on the ImageNet dataset (Krizhevsky et al., 2017). The question feature extraction models mainly include long short-term memory (LSTM) (Hochreiter and Schmidhuber, 1997), Glavnoe Razvedivatelnoe Upravlenie (GRU) (Cho et al., 2014), skip-thought (Kiros et al., 2015), and Bidirectional Encoder Representation from Transformer (Bert) (Devlin et al., 2018).

The attention mechanism (Xu et al., 2015; Shih et al., 2016) was introduced to implement model reasoning. Fukui et al. (2016) proposed to learn visual attention to image regions from questions and learn textual attention to question keywords. However, this local attention cannot reflect the similarity between image and question representations. Kim et al. (2016) indicated that the co-attention (Lu et al., 2016) of learning images and questions simultaneously facilitates fine-grained inter-modal representation, enabling more accurate predictions. However, co-attention learns only the coarse interactions between modalities and cannot derive the correlation between each image region and question keywords. The Stacked Attention Network (SAN) model (Yang et al., 2016) was first proposed to learn the attention of question-guided image regions by multi-word iterations, with each attention mechanism observing a different region of the image. Subsequent studies proposed a global attention strategy based on multi-glimpse attention (Fukui et al., 2016; Kim et al., 2016). For example, Lu et al. (2016) proposed the co-attention of image regions guided by question features, which requires not only learning visual attention to images but also textual attention to questions. Yu et al. (2018) simplified co-attention into two parts: one for self-attention learning from questions and another for cross-attention learning from question-guided images. Therefore, there is an inter-modal attention relationship between the image and question; however, existing methods are weak in fusing multimodal features owing to their neglect of inter-modal interactions.

With respect to multimodal feature fusion, the bilinear model encodes full second-order interactions that model the interaction between the two embedding spaces. Multimodal Compact Bilinear (MCB) pooling (Fukui et al., 2016), Multimodal Low-Rank Bilinear (MLB) (Kim et al., 2016), and Multimodal Tucker Fusion for Visual Question Answering (MUTAN) (Ben-Younes et al., 2017) are existing VQA methods for encoding images and questions using the bilinear transformation. They perform remarkably well in feature fusion, but the high dimensionality of the output features and the large number of model parameters may seriously limit the applicability of bilinear pooling. Therefore, some works should be done to simplify the bilinear model by reducing its complexity.

In this work, a new and effective VQA architecture was designed for fruit tree disease decision-making. This work includes (1) generating an image–question–answer triplet VQA dataset, including near-ground images, Unmanned Aerial Vehicle (UAV) remote sensing images, and Q&A text for fruit tree such as litchi, longan, grape, and other; (2) extracting image features by ResNet-152 and extracting question features by skip-thought and pretrained Bert model; (3) proposing a new multimodal factor decomposition bilinear pooling approach to effectively combine multimodal features, reducing the parameters from bilinear interactions between multimodal features through modal tensor decomposition; and (4) developing a co-attention mechanism with an end-to-end deep network architecture to jointly learn image and question attention to achieve model reasoning capabilities. The proposed method can provide fine-grained identification and decision-making for fruit tree diseases and provide a reference for farmers and companies in planning disease management and fertilizer application.

## 2 Materials and methods

### 2.1 Data acquisition and processing

Most image data in this study were collected in litchi orchards (23.55 N, 113.59 E) in Conghua and Zengcheng District, citrus orchards (23.15 N, 113.35 E) at South China Agricultural University, and other orchards. A total of 8,450 original images were collected through field research and photography. The text data of Q&A were mainly annotated manually under the guidance of agricultural experts and some websites corresponding to images of fruit tree diseases. The (image, question, answer) triplet for fruit tree diseases was constructed using the VQA dataset as a baseline, where each image was annotated manually with two questions, one question was annotated with three annotators, and all images were annotated with ground-truth answers. Specifically, each question was manually annotated after images and questions were accumulated, and for multiple choice questions, there were four candidate answers for each question. Four unique answers were collected that are correct, reasonable, universal, and random. Considering the redundancy of the answers, we initially set the number of annotated answers to 10 before the experiment. Due to a large amount of data annotation and most of the answers are apparent, the three most controversial and unbiased answers were eventually selected for annotation.

**Figure 2:**
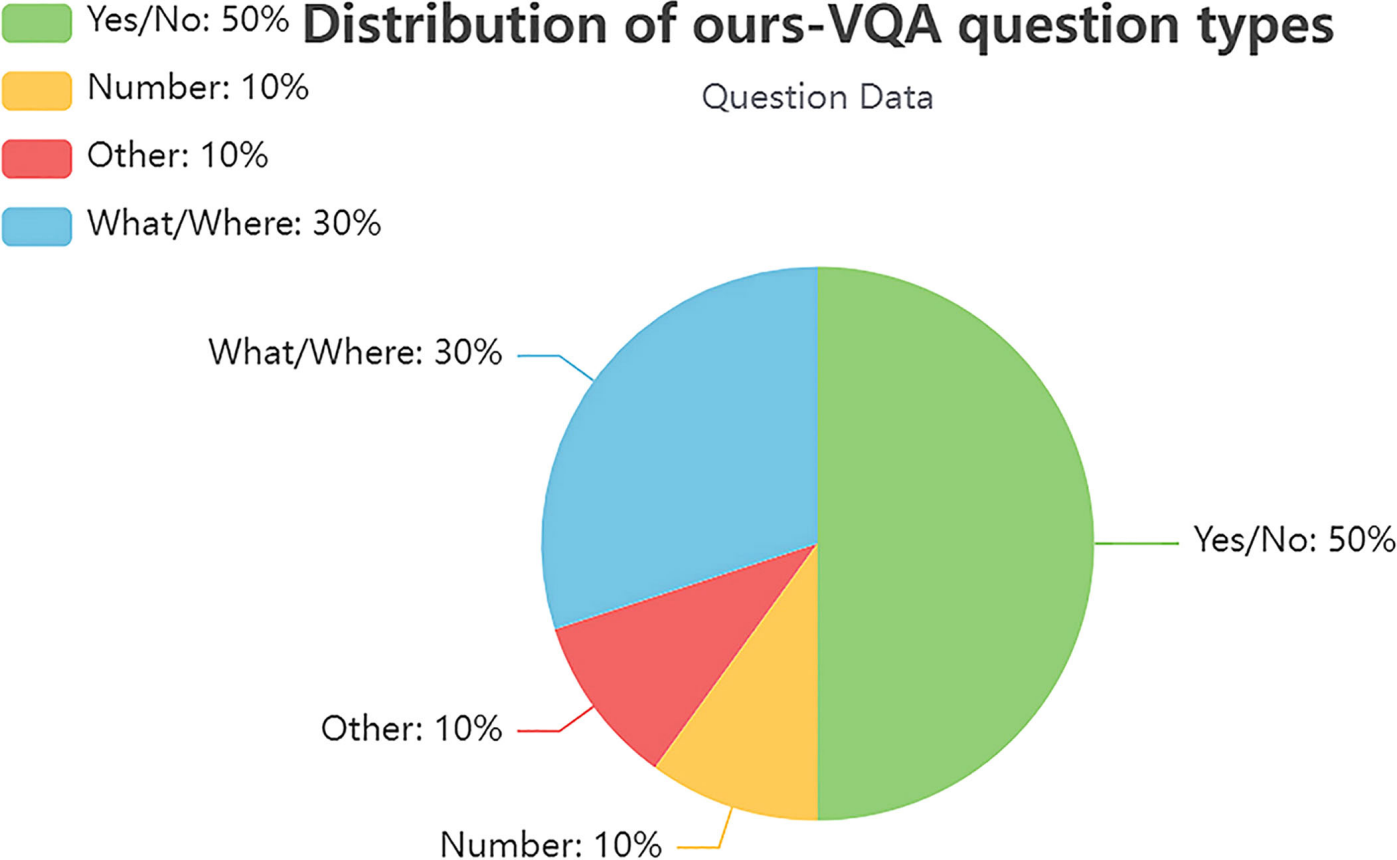
Data distribution of question types in the training set.

The dataset was divided into training set, validation set, and test set, in the ratio 8:1:1 (as **Table 1**): train (6,750 images and 13,500 questions), val (850 images and 1,700 questions), and test (850 images and 1,700 questions). The test set does not include annotated correct answers. The question–answer pairs include reasoning questions, colors, varieties, counts, and yes/no questions about diseases, including lychee fungal mildew, lychee anthracnose, citrus Huanglongbing disease, longan disease, and grape rot. As shown in **Figure 2**, question types were divided into four categories: yes/no questions, counting questions (how many), inference questions (what/where), and other. The study focused on multiple-choice questions, yes/no questions, and open-ended questions. The main purpose of the model is to answer the multi-categorization questions correctly about the fruit tree disease images in the test set.

**Table 1:** Statistics of the data.

	Training	Validation	Test
Images	6,750	850	850
Questions	13,500	1,700	1,700
Answers	40,500	5,100	0

To evaluate the accuracy of the model properly, we ensured that the results contained three main types of question accuracy (Yes/No, Number, Other, and What/Where) and overall accuracy. For easier reproduction in subsequent work studies, we save our dataset to Baidu.com and provide the download link of the dataset on: https://github.com/guoyaqi1/vqa_Fruit-tree-disease.

### 2.2 Data augmentation

To prevent overfitting or non-convergence of the model due to insufficient training data, we adopt data augmentation on images and tokenization on texts. Data augmentation (DA) (Zhang et al., 2021) can also increase the generalization ability of the model. Random data augmentation was adopted to augment the original image data. Random cropping, brightness adjustment, image rotation, ratio change, and random noise operations were performed to augment the image training dataset (Huang et al., 2019). Specifically, the original training image data are augmented with five methods. The first method is random image cropping to remove edge redundant information, with one random crop at edges 0 to 0.1 and 0.9 to 1. The second method is image rotation, which performs one random rotation at a rotation angle from 3° to 10°. The third method is the image ratio change, which scales the image aspect ratio once from 0.8 to 1.5. The fourth method is to inject random noise, creating a new noise-contaminated image for each image at random, using a zero-mean Gaussian noise with the mean and variance of noise injection set to 0 and 0.01. The fifth method is to adjust the brightness to 0.5–1.5 for one random brightness adjustment. For each data augmentation, five new images are generated for each image. All hyperparameters chosen here are empirical, which are used in the open published literature (Zhang et al., 2019; Wang et al., 2021b; Wang et al., 2021a), as shown in **Table 2**.

**Table 2:** The hyperparameter values of the image data augmentation.

Operation name	Description	Range of magnitudes
Random crop	Cropping of images by a range.	(0, 0.1), (0.9, 1)
Brightness	Adjust the brightness of the image.	(0.5, 1.5)
Ratio change	Scaling the width and height of an image.	(0.8, 1.5)
Image rotation	Rotate the image magnitude degrees.	(3, 10)
Random noise	Injecting noise into the image.	(0, 0.01)

For texts, all questions and answers are converted into lower case letters and remove the punctuation marks and question samples by replacing words, deleting words, text noise addition, and sampling-based methods (Ren and Zhou, 2020). Since word-based tokens will cause a large dictionary, we used the word piece tokenization method, like the Bert model.

## 3 Multimodal network architecture

**Figure 3:**
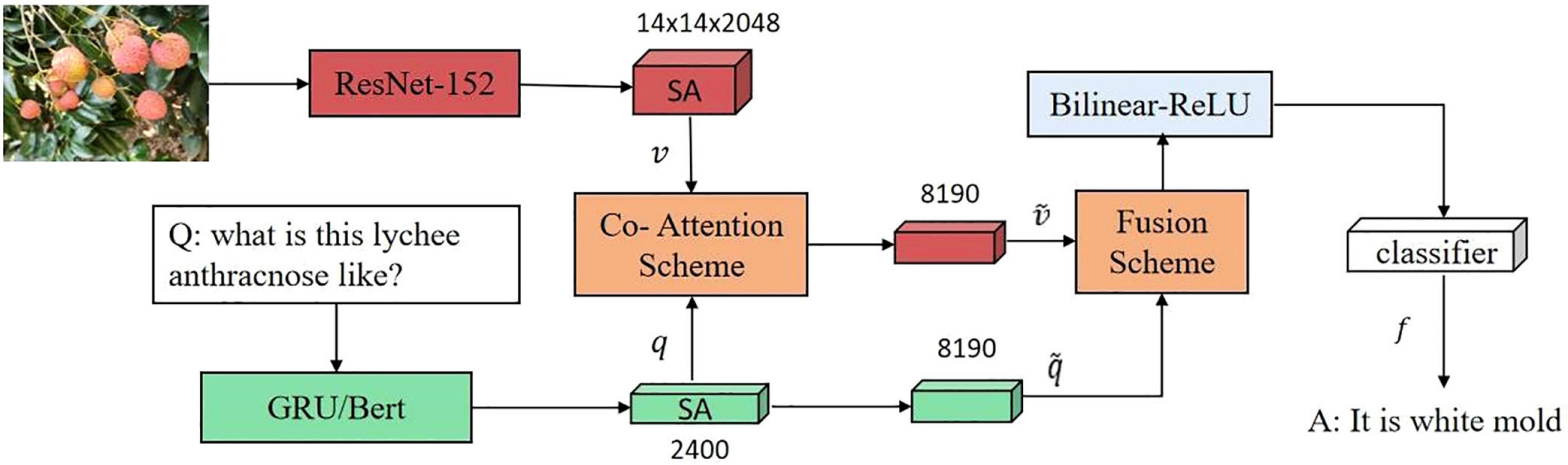
The proposed framework of VQA.


In this work, a new VQA architecture for fruit tree disease decision-making was proposed. The proposed framework of VQA is shown in **Figure 3**.** **A pretrained network was adopted to first extract the image 144 features v and the question features *q*; then, a novel fusion scheme with multiple co-attention layers was employed to learn inter-modality relations, which fuses visual features first and then the textual features. The fused features obtained from the last co-attention layer is used for fruit tree disease decision. In particular, a simple but powerful image-centric scheme that emphasizes the image was proposed. The obtained vector of potential features of dimension N = |*A*| is activated by Softmax and used to predict the most likely answer. As with existing VQA methods, the goal in this study was to predict the most likely answer, $\hat{a}$ , to a question, *q* , about an image, *v* . The problem can be formulated as Equation (1):

(1) $$\hat{a}=\underset{a\in A}{\arg\;\max\;}P(a|q,v,\text{Θ})$$

**Figure 4:**
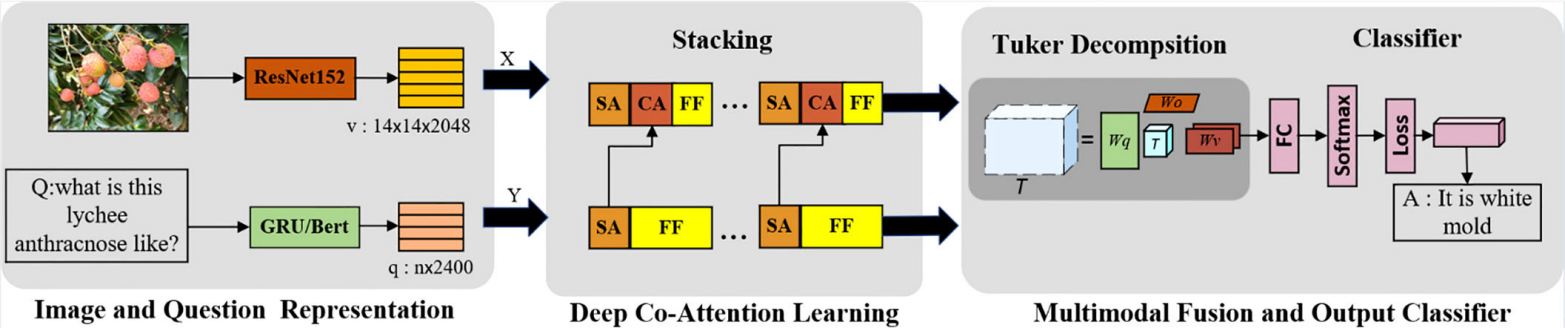
The proposed VQA model architecture together with our multimodal deep co-attention network.

Where *â* is the set of possible answers, and Θ contains all model parameters. The model is divided into three core learnable elements: (i) an image model that extracts visual features from the input images and a question model that encodes the question input; (ii) an attention mechanism that finds important regions in the input images; and (iii) a fusion mechanism with bilinear Tucker decomposition, which combines visual and question features. Finally, the model requires a classifier that selects the highest scoring answer among a set of candidate answers. The architecture of the proposed VQA model is shown in **Figure 4**.

### 3.1 Image and question model

**Figure 5:**
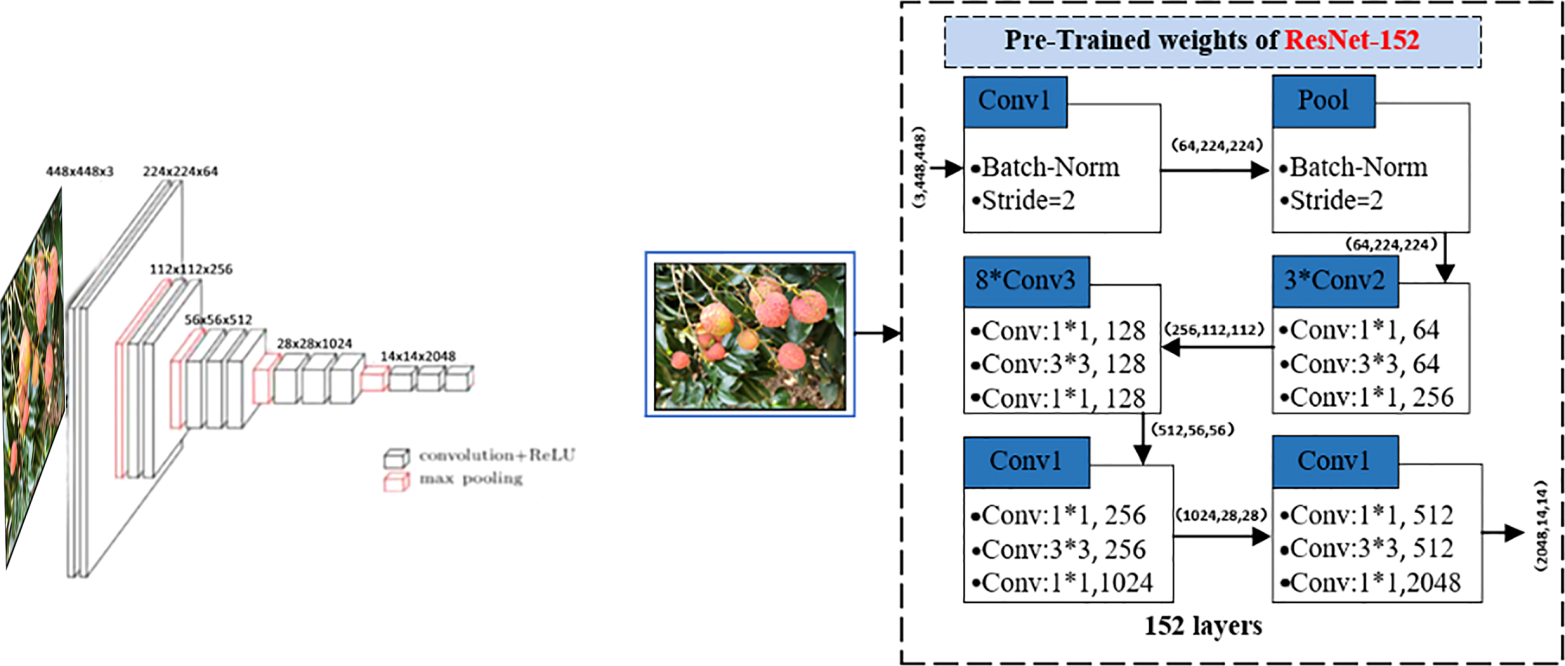
The ResNet-152 model for image features extraction.

The preprocessing pipeline and data augmentation were first applied to the image data. To erase unnecessary external information (image regions and text) from the image, we normalized the intensity of the input image to 0–255 and set the threshold value of the normalized image to 5 by applying the Otsu method (Otsu, 1979), used to threshold the image based on the difference in grayscale between the target region and the background to be extracted in the image, and selected the best threshold to determine whether the add feature attribute of each pixel point belongs to the target region or the background. An open operation was applied to the post-threshold image, which has a rectangular structuring element of size 40 × 40. After calculating a foreground bounding box, the image was cropped to the bounding box, the cropped image was resized to 448 × 448, and the resized image was inputted to ResNet-152 model to extract image features to obtain 2048-D vector output, as shown in **Figure 5** and **Table 3**.

**Table 3:** The ResNet-152 structure.

Layer name	Output size	Kernel size	Channel	Number
conv1	224 × 224	(7, 7)	64	1
conv2	112 × 112	(1, 1)	64	3
		(3, 3)	64	3
		(1, 1)	256	3
conv3	56 × 56	(1, 1)	128	8
		(3,3)	128	8
		(1,1)	512	8
conv4	28 × 28	(1, 1)	256	36
		(3, 3)	256	36
		(1, 1)	1024	36
conv5	14 × 14	(1, 1)	512	3
		(3, 3)	512	3
		(1, 1)	2048	3

The ResNet-152 model was adopted to extract the image features, we experimentally compared VGG, InceptionResNetV2 (Baldassarre et al., 2017), ResNet-50 (Akiba et al., 2017), and ResNet-152 (Fukui et al., 2016). ResNet-152 is the best performing neural networks in image feature extraction tasks. Different convolutional layers have different feature extraction capabilities. ResNet-152 has five blocks that resize the image by convolution kernels. The features of the image are extracted through the five convolutional layers of ResNet-152. The extracted feature sizes are shown in **Figure 5**. An untrained full convolutional network (Tong et al., 2021) was used to unify the number of feature maps and a global average pooling strategy was used to unify the sizes.

The Residual Unit structure is Conv-BN-ReLU-Dropout-Conv-BN-ReLU-Dropout–Conv-BN-ReLU, where BN is a batch normalization operation to maintain the same input distribution for each layer of the network, rectified linear unit (ReLU) is the activation function, Conv denotes the convolution layer, and dropout layers are added between the convolutional layers in each residual branch to prevent overfitting, where the dropout ratio is 10^-4^.

For the question representation, the question data were preprocessed using the same data preprocessing techniques used in Fukui et al. (2016) and Ben-Younes et al. (2017), mainly by deleting punctuation marks, converting the question characters to lowercase letters, and removing all combinations. The question was divided into a series of words using the space character. The pretrained skip-thought model was obtained by pretraining on the collected text dataset. A special “unknown” word (“UNK”) was used to state the case that does not exist in the dataset. Finally, after zero padding, the length of all the word sequences was 26 words, matching the maximum sequence.

To overcome the shortcoming of unknown or unseen new term words in the plant domain, we encoded unseen words in the model by transfer learning (Kiros et al., 2015). First, a Word2Vec model (Mikolov et al., 2013) trained on the Google News dataset (Das et al., 2007) was used, which contains a vector of 3 million words and phrases. Second, a weighted linear regression model was fitted by minimizing a least squares criterion, which maps Word2Vec to the skip-thought embedding space. This enabled the pretrained skip-thought model to generate 2,400 dimensions of question features. The above adopted skip-thought models can represent words as vectors for better training; global vectors (Pennington et al., 2014) map words into meaningful space based on semantic similarities. However, this method cannot disambiguate according to the context, since the vector of each word is fixed. Text-embedding model learns the context of words by deep networks. Compared with words vectors, the text-embedding model adjusts the word vector according to the context, but it requires a high computational cost (Wu et al., 2021).

The pretrained models like Bert has achieved a breakthrough result, which performs well on contextualized text representation. Bert was adopted to extract the question features, coping with mask text model tasks and next sentence prediction tasks at the same time. In the Bert model, the second-to-last hidden (768 dimensions) method was applied to generate a pretrained contextual representation similar to Embeddings from Language Models (ELMO) (Devlin et al., 2018). The pretrained Bert model can obtain the fine-grained feature sequence containing the contextual information so that the question sequence contains both its own information and the relationship with all the data.

### 3.2 Attention mechanism

As the simple attention mechanism cannot infer the correlation between the question keywords and the image regions, in this study, we proposed a co-attention mechanism and a self-attention and multi-head attention mechanisms based on the transformer architecture. First, we perform self-attention fusion for question and image features separately. Second, the output features of the two modalities are fed into the co-attention mechanism for interaction. Therefore, the attention module can mainly be divided into a self-attention (SA) unit and co-attention (CA) unit, combining into the modular co-attention (MCA) layer, which is capable of modeling the self-attention of questions and images and the question-image guided co-attention.

#### 3.2.1 Self-attention and co-attention units

**Figure 6:**
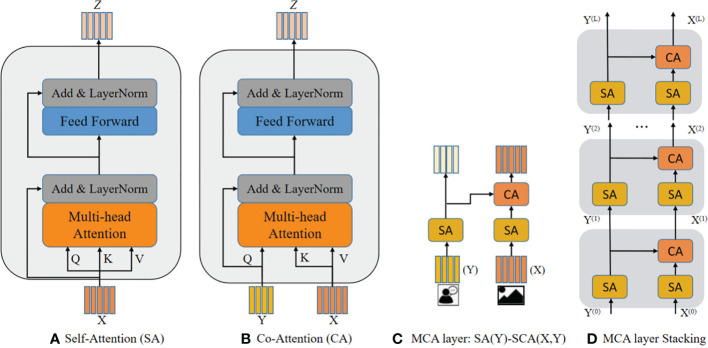
The comparison of SA, CA, and MCA **(A)** shows the components of the SA unit, **(B)** shows the improvement of the CA unit on basis of the SA unit, **(C)** shows the MCA module obtained by combining SA and CA, and **(D)** shows the model reasoning obtained by stacking the MCA modules.

The SA unit shown in **Figure 6A** consists of a multi-headed self-attention function and a fully connected feed-forward network, both wrapped in a residual connection followed by layer normalization. The CA unit is extended from the SA unit, as shown in **Figure 6B**. The keys and values are from one modality, while queries are from another modality, and the queries are used as a residual item after the multi-head attention sublayer. The rest of the architecture is the same as the SA unit. The CA unit takes the features of two modalities features, and one modality guides the attention learning for another modality. Assuming that Q comes from the question and K and V come from the corresponding images, the attention value calculated by Q and K can be used to measure the similarity between the question and the image and then weight the image. Importantly, co-attention can model the intra-modal interaction between different features.

#### 3.2.2 Multiple co-attention stacking

To deeply fuse multimodal features, we combine the two basic attention units to obtain a MCA layer to handle the multimodal features, which consists of two SA units and one CA unit, named the SA(Y)-SCA(X,Y), image features X and question features Y are used as inputs, as shown in **Figure 6C**. The MCA layer models the intra-modal interactions between each image region pair. We stacked multiple MCA layers to compose a deep co-attention model to deliver the input features and evaluate the depth layers, as shown in **Figure 6D**.

### 3.3 Bilinear fusion scheme

The issues caused by too many learning parameters in the fusion mechanism are twofold: (i) better graphics processing units (GPUs) are needed, and (ii) the VQA model learning process is prone to overfitting. A low-rank bilinear method (Pirsiavash et al., 2009) has been proposed to reduce the rank of the weight matrix. To reduce the large number of parameters generated by multimodal bilinear model interactions, in this study, we proposed a multimodal fusion scheme based on a bilinear pooling fusion model with Tucker decomposition based on the intermodal correlation tensor, which is not simply connecting two modalities (Ren et al., 2015). The tensor *T* in the bilinear fusion architecture is decomposed using a Tucker decomposition.

**Figure 7:**
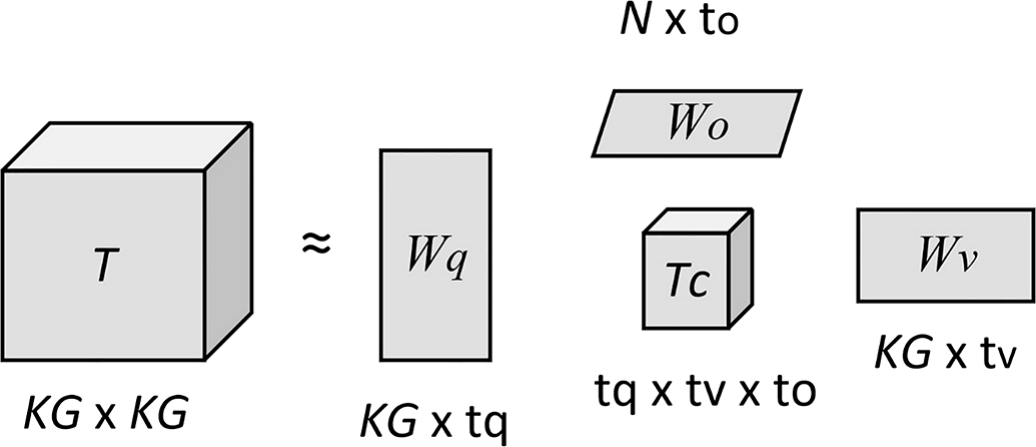
The illustration of Tucker decomposition.

In this fusion scheme, the *q*∈*ℝ* ^*J*^ is the question feature, *q*∈*ℝ* ^*KG*^ is the image feature, and *f*∈*ℝ* ^*N*^ is the answer feature corresponding to *a*∈*A* . K=2,048 and J=2,400 are the inputs to the image and question, respectively, and K is the dimension of the core tensor of the constant equation. G denotes the number of multimodal attention, $W_{Kg}^{e}\in {\mathbb{R}}^{KG\times J}$ is the weight matrix of features under attention mechanism, and $B_{Kg}^{e}\in \mathbb{R}$ is a bias item. Before fusing features, transformation must be performed, as shown in **Figure 7**.

The decomposition of the tensor of a three-way tensor *T*∈*ℝ* ^*d*_*q*_^×*d*^_*v*_^×^|*A*|^ is expressed as a tensor product between three intra-modal factor matrixes: *W*_q_ , *W*_v_ , and *W*_o_ , and a core tense *T*_c_ in Equation (2):


(2) $$T=\left(\left(T_{c}\times_{1}W_{q}\right)\times_{2}W_{v}\right)\times_{3}W_{o}$$

where

*W*_q_∈*ℝ* ^*d*_*q*_×*t*_*q*_^,

*W*_v_∈*ℝ* ^*d*_*v*_×*t*_*v*_^,

*W*_o_∈*ℝ* ^|*A*|×*t*_*q*_^, and


$$T_{c}\in {\mathbb{R}}^{t_{q}\times t_{mathrmv}\times t_{\text{o}}}$$

The probability that each target answer is on all possible target answers is in Equation (3):


(3) $$f=\left(T\times_{1}{\tilde{q}}^{T}\right)\times_{2}{\tilde{v}}^{T}+b^{f}$$

We combine Equations (2) and **(3)** such that:


(4) $$f=\left(\left(T_{c}\times_{1}\left({\tilde{q}}^{T}W_{\tilde{q}}\right)\right)\times_{2}\left({\tilde{v}}^{T}W_{\tilde{v}}\right)\right)\times_{3}W_{o}$$

In this study, we found that squared transformations on image features improved the attention of the model to image features and reduced the linguistic bias of the model. Inspired by MUTAN (Ben-Younes et al., 2017), we proposed a simple and efficient extension. The improved fusion model yields Equation **(5)**.

**Figure 8:**
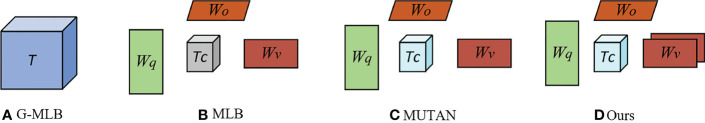
A comparison of VQA fusion models. **(A)** G-MLB (Vu et al., 2019): full tensor is trainable and is not decomposed like other methods. **(B)** MLB: Wq, Wv, and Wo are trainable, while Tc is fixed. **(C)** MUTAN: all four elements are trainable. **(D)** Our model: similar to MUTAN, with proposed element-wise square of Wv; the full bilinear interaction is structured with a low-rank (R) decomposition.


As most VQA studies only obtain prediction answers through questions, this study proposed an image-centered model by emphasizing image features, the comparison of VQA fusion schemes is shown in **Figure 8**.

(5) $$f_{\text{vcmut}}=\left(\left(T_{c}\times_{1}\left({\tilde{q}}^{T}W_{\tilde{q}}\right)\right)\times_{2}{\left({\tilde{v}}^{T}W_{\tilde{v}}\right)}^{2}\right)\times_{3}W_{o}$$

### 3.4 Multimodal fusion and output classifier

After the deep co-attention learning stage, the output image features and question features already contain rich information about the attention weights on the question words and image regions. First, we designed a bilinear multimodality to fuse the image and question features *◯* and *ŷ* after the modular attention stage, and the bilinear function is defined as the Equation (6):


(6) $$z=LN\left(W_{x}^{T}\tilde{x}+W_{y}^{T}\tilde{y}\right)$$

where *W*_*x*_, *W*_*y*_∈R^d× d*z*^ are bilinear projection matrices, and *z* is the fused multimodal feature that is used to predict the answer. d*z* is the output dimension of the fused features. Layer normalization (*LN* ) is used here to stabilize training. We designed the final fully connected layer with the output dimension n and connect the softmax layer for n classification prediction, where the loss function is the categorical cross-entropy. The evaluation metric is strict accuracy.

## 4 Experimental results

### 4.1 Experiment model setting

The proposed fusion model mainly adopts a co-attention mechanism and a bilinear pooling fusion mechanism using multi-head attention of 8 and a hidden layer size of 512. To balance accuracy and information loss, we set the maximum input length of the text to 26. We set the dimension to 2,048 in the bilinear pooling, which works best for the visual grounding task. The layer normalization before the attention and feedback layers is set with *L*_2_=1×10^−12^ .

In the optimization process, we replaced all hyperbolic tangent (tanh) activations with ReLU activation functions. The network for fruit tree disease was trained from a random initialization with the AdamW (Kingma and Ba, 2014; Loshchilov and Hutter, 2017) optimizer, with a learning rate of 0.0001. We compared SGD, Adam, and AdamW, among them, AdamW performed the best. Dropout rate was 0.5 for all linear and bilinear layers, learning rate decay was *β* 1 = 0.9 and *β* 2 = 0.999, and the mini batch size was 64. Moreover, early stopping was used as a regularization to save model parameters after each epoch to prevent overfitting.

During the training process, early stopping was used as a regularization to save model parameters after each epoch to prevent overfitting. To evaluate the model, we chose the best epoch based on the accuracy of the validation set.

The proposed method was trained using the PyTorch library, and the experiments were run on Nvidia GTX3090 Ti 32GB GPU. The implementation is available at https://github.com/guoyaqi1/vqa_Fruit-tree-disease. After tuning all model parameters by training, we trained the model once on all available data (training set + validation set). Finally, we evaluated the test set to obtain the evaluation results of the model. The main hyperparameter setting is shown in **Table 4**. Most of the values are set by trial-and-error method, and **Table 5** shows the hyperparameters setting in this study.

**Table 4:** Setting of important parameters.

Modality	Parameter name	Parameter value
Text	Bert-base-cased	768
	skip-thought	2,400
	LSTM	1,024
	Max length	26
Image	Image sizes	448 × 448
	Output dimensions	2,048
Other	Dropout	0.5
	Batch_size	100
	lr	0.01
	Loss	CrossEntropyLoss
	Optimizer	AdamW
	Activation	RELU

**Table 5:** The experiments of setting the hyperparameters.

Hyperparameters	Accuracy
Reference model	0.86
Reference: hidden size=512	
Hidden size=312	−0.006
Hidden size=600	+0.003
Reference: embedding size=500	
Embedding size=440	−0.008
Embedding size=hidden size	+0.006
Reference: heads=8	
Heads=6	−0.012
Reference: prelayer normalization	
Post-layer normalization	−0.004
Reference: no dropout	
Dropout=0.5	+0.002
Reference: no early stopping	
early stopping	+0.010
Reference: learning rate=0.0001	
learning rate=0.1	−0.010
Reference: batch size=64	
Batch size=128	−0.012
Batch size=32	−0.020

### 4.2 Comparison of fusion schemes

Under the same experimental setting, the proposed model was evaluated and compared with three bilinear models (MCB, MLB, and MUTAN) without using the attention model. The comparison of fusion schemes results are shown in **Table 6**: Concat denotes a baseline where *v* and *q* merged by simply concatenating unimodal features without considering inter-modality relations. **Table 6** shows that the proposed model performed better than other bilinear fusion models, which validates the idea that modeling full bilinear interactions between low-dimensional projections yields a more efficient representation than a strong unimodal transformation with simple fusion scheme. Furthermore, there was a well-balanced trade-off between the projection dimension in the core tensor *Tc* and the bilinear interaction parameters. The last row in **Table 6** presents the proposed model with our attention mechanism, which obtained the best result, validating the idea that the proposed attention mechanism effectively outperforms other bilinear fusions.

**Table 6:** Comparison of fusion schemes with no_att.

Fusion	Question	Image	Activation	Batch_size	Epoch	Acc
Concat	Skip-thought	ResNet-152	tanh	128	100	60.10
MCB	Skip-thought	ResNet-152	tanh	128	100	70.01
MLB	Skip-thought	ResNet-152	tanh	128	100	70.12
MUTAN	Skip-thought	ResNet-152	tanh	128	100	81.15
Proposed	Skip-thought	ResNet-152	tanh	128	100	**82.15**
Proposed	Skip-thought	ResNet-152	RELU	128	100	**82.36**
**Proposed+myatt**	Skip-thought	ResNet-152	RELU	128	100	**86.36**

Bold values emphasize that the accuracy obtained by the improved model in the article achieves better results than the other models.

### 4.3 Ablation experiments

#### 4.3.1 Attention mechanism

The evaluation of the impact of the attention mechanism on model performance for four kinds of questions is shown in **Table 7**, where I denotes the image encoding module, Q denotes the question encoding module, A denotes the attention mechanism, NA denotes no attention mechanism, SA denotes that the image or question module contains a self-attention mechanism, CA denotes modeling dense interactions between input modalities by exchanging their information, and MCA denotes the combination of SA and CA to obtain our proposed attention fusion mechanism. Specifically, SA is used in the image and question encoding modules separately and the model with CA in the feature fusion stage, named (Q(SA) + I(SA)) CA in **Table 7**.

**Table 7:** Attention mechanism comparison.

Model	All	Y/N	Num	What/Where	Other
Q(NA)+I(NA)	82.15	90.13	68.23	70.82	70.85
Q(NA)+I(SA)	82.98	90.58	68.82	71.21	71.26
Q(SA)+I(NA)	82.78	90.34	68.72	71.01	71.08
Q(SA)+I(SA)	84.25	91.32	69.36	72.83	72.90
(Q(NA)+I(NA))CA	85.36	92.21	69.95	73.51	73.53
(Q(SA)+I(NA))CA	86.21	93.19	70.21	74.13	74.18
**(Q(SA)+I(SA))CA**	**86.36**	**93.38**	**70.56**	**74.34**	**74.34**

Bold values emphasize that the accuracy obtained by the improved model in the article achieves better results than the other models.

**Table 7** shows that MCA obtained the best results, proving that the MCA attention mechanism can improve the interaction of image and text multimodal feature fusion. It shows that (Q(SA) + I(SA)) CA outperformed (Q(SA)+I(NA))CA, which illustrates that modeling self-attention for image features is valuable. Moreover, the Q(NA)+I(SA) also outperformed Q(SA)+I(NA) for all question types, which verifies that modeling self-attention for image feature benefits model performance. The above results show that the contribution of the image module is more significant than that of the question module, validating the idea of the proposed Tucker decomposition method to fuse features in an image-centric manner, which reduces the model parameters while also increasing the weight of the model on the image features.

#### 4.3.2 Depth of MCA

**Figure 9:**
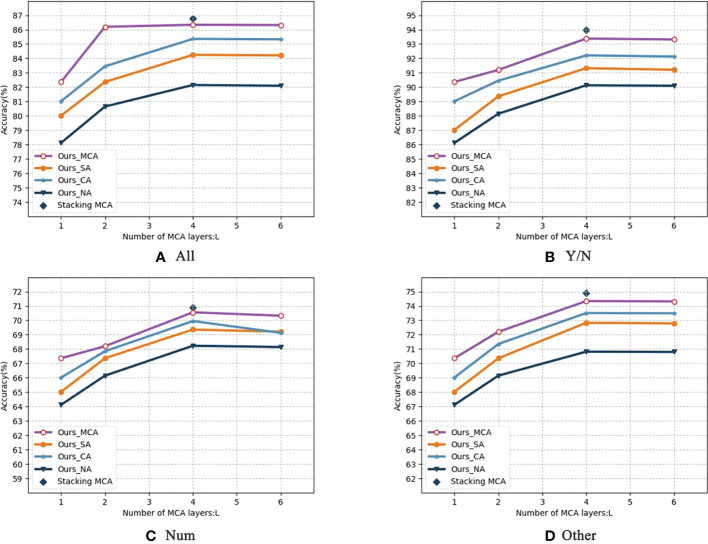
Comparison of four attention modules in depth(All&YES/NO&Num&Other). It shows the detailed performance of MCA- L with different attention modular under per-type questions. With increasing L, the performance gaps between the four attention modular increased.

**Table 8** shows the impact of the number of MCA layers *L* for the stacked attention module on the performance, setting *L*= 1,2,4,6} and model sizes (number of parameters). The results in **Table 8** shows that the performance of the deep co-attention models steadily improved and finally saturated at *L* = 4 with the increase of *L*. Therefore, *L* = 4 is the best setting considering the optimized performance and reduced resource overhead. **Figure 9** shows the detailed performance of MCA- *L* with different attention modular under per-type questions. With increasing *L*, the performance gaps between the four attention modular increased. This validates that the depth of MCA layers for images plays a key role in the model.

**Table 8:** Depth MCA layer L stacking.

L	MCA-acc	Size
1	82.36	27M
2	86.32	41M
4	**86.36**	56M
6	86.34	68M

Bold values emphasize that the accuracy obtained by the improved model in the article achieves better results than the other models.

#### 4.3.3 Question representation

**Table 9** shows the results of the ablation experiments with different question representations on MLB, MUTAN, and the proposed model. Considering the text encoding model for extracting question features, there are three question representation models (“Bert-base-uncased,” “Bert-base-cased,” and skip-thought) were used for comparison, and all fusion schemes with attention mechanisms were used for all models. In **Table 9**, *J* denotes the dimension of the question feature space. **Table 9** shows that using tokens by pretrained Bert significantly outperformed the skip-thought vectors and Bert-base-uncased, which indicates that the pretrained Bert model is more effective in extracting question features.

**Table 9:** Accuracy of the proposed models with different question representations.

Model	Question	*J*	All	Y/N	Num	What/Where	Other
MLB_att	Bert-base-cased	2400	71.60	80.23	60.21	68.85	68.75
	Bert-base-uncased	768	70.34	80.12	60.17	68.79	68.71
	Skip-thought	768	70.21	80.10	60.03	68.67	68.65
MUTAN_att	Bert-base-cased	2400	83.36	90.23	65.87	72.23	72.25
	Bert-base-uncased	768	82.57	90.19	65.81	72.15	72.12
	Skip-thought	768	82.13	90.03	65.68	72.13	72.09
Proposed_att	Bert-base-cased	2400	**86.36**	**93.38**	**70.56**	**74.41**	**74.34**
	Bert-base-uncased	768	85.52	93.21	70.48	74.32	74.31
	Skip-thought	768	85.36	93.35	70.41	74.29	74.28

Bold values emphasize that the accuracy obtained by the improved model in the article achieves better results than the other models.

### 4.4 Cross-validation

**Figure 10:**
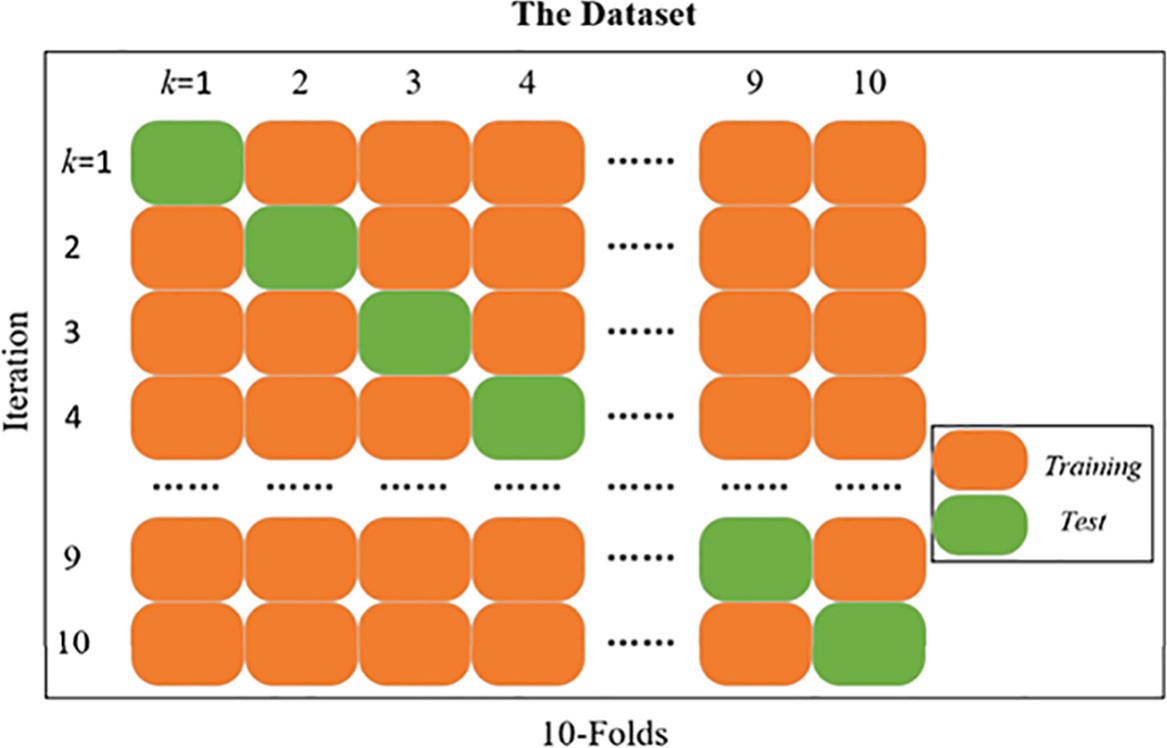
Diagram of the 10-fold cross-validation.

Cross-validation (Albashish et al., 2021) is a way of resampling a dataset to evaluate algorithmic models on limited size dataset (Wang et al., 2021b). Cross-validation reduces the coincidence due to previous random divisions by splitting the dataset multiple times, makes the algorithm accuracy fairer, and improves the generalization of the model. **Figure 10** shows the diagram of the k-fold cross validation. Specifically, the whole dataset is divided into K folds evenly. For the *k*th (k=1,…,K) trial, the *k*th fold is used for the test set and the other folds (1,…,k−1,k+1,…,K) for training. In this study, the 10-fold cross-validation was adopted to validate the accuracy of model. It performs 10 iterations of the experiment by splitting the dataset into 10 parts and rotating nine of the dataset as the training set and one as the test set.

### 4.5 Statistical analysis

The results of 10 runs of 10-fold cross-validation of our model are itemized in **Table 10**. The classifier evaluation on yes–no question types was performed using statistical tests, and we selected five evaluation indicators from the confusion matrix: sensitivity, specificity, precision, accuracy, and F1 score. The mean and standard deviation (mean+SD) (Zhang et al., 2021) of all five indicators are calculated over 10 runs. The statistical results of proposed model on yes–no classifier are shown in **Table 6**. The sensitivity and specificity reached 93.48 ± 1.27 and 93.28 ± 1.45, respectively. Its precision and accuracy are 93.29 ± 1.46 and 93.38 ± 1.37, respectively. The F1 score is obtained as 93.37 ± 1.36. As a result, the differences between the algorithms are statistically significant.

**Table 10:** Statistical results of proposed model on yes–no classifier.

Run	Sensitivity	Specificity	Precision	Accuracy	F1 score
1	91.25	93.11	92.98	92.12	92.23
2	94.29	92.39	93.38	93.34	93.34
3	92.04	93.73	92.53	93.09	92.42
4	93.10	91.38	91.24	92.24	92.23
5	94.93	94.19	94.09	94.56	94.55
6	93.69	92.09	92.21	92.89	92.88
7	94.12	92.34	92.47	93.23	93.22
8	94.55	96.35	96.28	95.45	95.44
9	91.75	92.75	92.66	92.15	92.15
10	95.08	94.38	95.06	94.73	95.24
Mean ± sd	**93.48** ± **1.27**	**93.28** ± **1.45**	**93.29** ± **1.46**	**93.38** ± **1.37**	**93.37** ± **1.36**

Bold values emphasize that the accuracy obtained by the improved model in the article achieves better results than the other models.

### 4.6 Attention visualization

**Figure 11:**
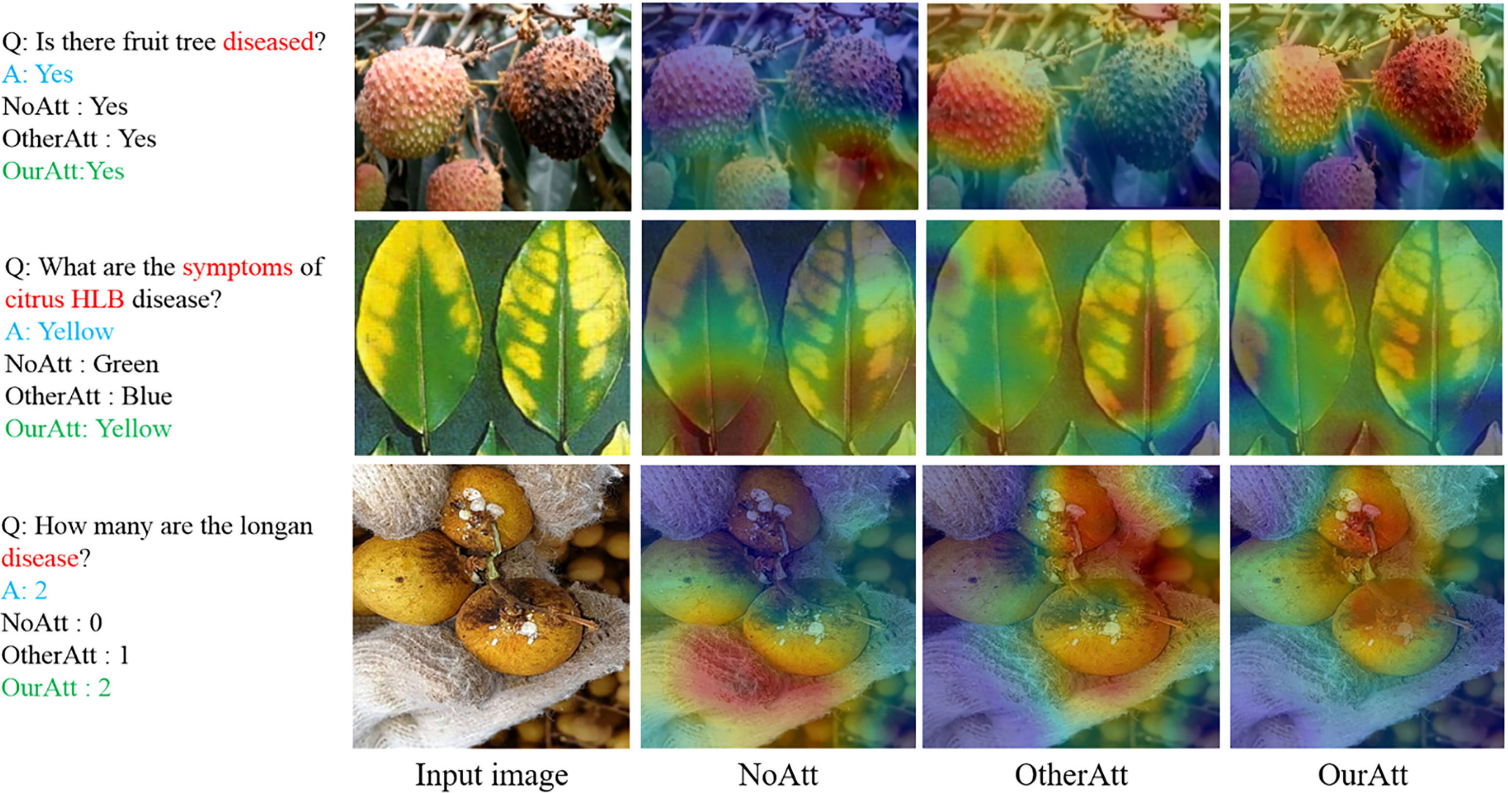
Attention visualization. The first column is the original image of the input, the second column is the image region computed by the model under no attention (NoAtt), and the third and fourth columns are the image region computed by the model under other attention (OtherAtt) and its own attention (MyAtt), respectively.

**Figure 11** shows the visualization of the attention learning from the questions and the images. The text on the left of **Figure 11** shows Q&A including the ground truth and the results from the proposed model without attention (No. Att.) and with attention (OtherAtt. &MyAtt) mechanisms. The red area indicates the image region on which the model is focused by attention. It can be observed that the model with the attention mechanism produced a more focused localization area for the disease presented by the question compared with the model without attention. While using our proposed attention scheme, the relevant regions in the input image are highlighted to a greater extent. For example, the first question in the figure is “Is there fruit tree diseased?”. The model focused on the diseased area under our proposed attention scheme. This indicates that learned question attention focuses more on keywords, and learned image attention focuses more on the correlation between keywords and corresponding image regions.

## 5 Discussion

The abovementioned experiments showed that the proposed VQA model of fruit tree disease was superior to other existing multimodal methods combining an optimized bilinear model with stacking MCA layers. The proposed model achieved high performance for the following main reasons: first, a stacking modular co-attention (MCA) layer for multimodal interaction of images and questions makes the model more capable of learning effective features. Neither self-attention nor co-attention can infer the correlation between each image and each problem separately, so the mutual synergy between self-attention and co-attention, that is, the simultaneous learning of image and question co-attention, is more beneficial for fine-grained feature representation of images and questions. The appropriate depth of the MCA layer can provide more fine-grained extraction and enhance model reasoning capabilities for feature fusion. Second, image and text features are transformed using bilinear pooling instead of inner product operation, and the Tucker decomposition of tensor during fusion makes the parameters of bilinear interaction controllable. Finally, the question features extracted by the pretrained Bert models perform a little better than those produced by the skip-thought vectors, enabling the model to obtain a better question representation. Since this study is a multimodal fusion multiclassification issue, the final statistical test and 10 times 10-fold cross-validation on yes or no question types yielded statistically significant results for the algorithm.

However, there were some limitations to this study. For example, inaccurate positioning of keywords led to incorrect prediction answers. In addition, from attention visualization, it could be found that attention learning was stochastic in the experiments, sometimes, it failed to distinguish keywords in the questions, resulting in focusing on irrelevant image regions and false predictions. These visualization results can help us make further improvements to the model.

In future precision agriculture decision-making, dataset optimization should not only consider visible images and Q&A pairs but also increase the text information of agricultural expert knowledge in the agricultural knowledge map to improve the reasoning of the model.

## 6 Conclusion

In this work, we proposed a new multimodal attention network for VQA of fruit tree disease. The model is mainly divided into four modules: image feature extraction, question feature extraction, feature fusion with attention mechanism, and a bilinear fusion model. The main contributions were the co-attention modularity to interact with multimodal information by stacking MCA layers and the bilinear pooling fusion model combining a Tucker decomposition with a low-rank matrix constraint. The experiments showed that the proposed VQA model outperformed other state-of-the-art methods. The average accuracy of the proposed VQA model with stacking MCA layers reached 86.36%, outperforming other bilinear fusion methods; the optimum depth of the MCA layer was 4, and the pretrained Bert outperformed the skip-thought in extracting question features. This work provides in-depth insights for VQA in the field of plants and provides a way to greatly reduce human labor resources and implement effective artificial intelligence applications in agriculture.

## Data availability statement

The raw data supporting the conclusions of this article will be made available by the authors, without undue reservation.

## Author contributions

YL conceptualized the experiment, wrote the original draft, and performed funding acquisition. YG conceptualized the experiment, selected the algorithms, collected and analyzed the data, and wrote the manuscript. QC trained the algorithms, collected and analyzed data, and wrote the manuscript. SL and YC carried out the experiments. XD supervised the project and funding acquisition. All authors discussed and revised the manuscript. All authors contributed to the article and approved the submitted version.
